# Cellulitis caused by *Roseomonas mucosa* in a child: a case report

**DOI:** 10.1186/s12879-023-08875-9

**Published:** 2023-12-11

**Authors:** Yuki Matsuhisa, Tsuneaki Kenzaka, Hideo Hirose, Tadao Gotoh

**Affiliations:** 1 Department of Pediatrics, Center for Community Medicine in North-Western Gifu Prefecture, National Health Insurance Shirotori Hospital, 1205-1, Tamezani, Shirotori-cho, Gujo, Gifu, 501-5122 Japan; 2 Department of General Medicine, Center for Community Medicine in North-Western Gifu, Prefecture National Health Insurance Shirotori Hospital, 1205-1, Tamezani, Shirotori-cho, Gujo, Gifu, 501-5122 Japan; 3 Department of Internal Medicine, Hyogo Prefectural Tamba Medical Center, 2002-7 Iso, Hikami- cho, Tamba, Hyogo 669-3495 Japan; 4 Division of Community Medicine and Career Development, Kobe University Graduate School of Medicine, 2-1-5 Arata-cho, Hyogo-ku, Kobe, Hyogo 652-0032 Japan

**Keywords:** *Roseomonas mucosa*, Cellulitis, Child, Asteatotic eczema, Steroid ointment

## Abstract

**Background:**

*Roseomonas mucosa* (*R. mucosa*) is a pink-pigmented, Gram-negative short rod bacterium. It is isolated from moist environments and skin, resistant to multiple drugs, including broad-spectrum cephalosporins, and a rare cause of infection with limited reports. *R. mucosa* mostly causes catheter-related bloodstream infections, with even fewer reports of skin and soft tissue infections.

**Case presentation:**

A 10-year-old boy received topical steroid treatment for sebum-deficient eczema. A few days before the visit, he was bitten by an insect on the front of his right lower leg and scratched it due to itching. The day before the visit, redness, swelling, and mild pain in the same area were observed. Based on his symptoms, he was diagnosed with cellulitis. He was treated with sulfamethoxazole/trimethoprim, and his symptoms improved. Pus culture revealed *R. mucosa*.

**Conclusions:**

We report a rare case of cellulitis caused by *R. mucosa*. Infections caused by rare organisms that cause opportunistic infections, such as *R. mucosa*, should be considered in patients with compromised skin barrier function and regular topical steroid use. Gram stain detection of organisms other than Gram-positive cocci should be considered.

## Background

The genus *Roseomonas* is a slow-growing Gram-negative coccobacilli with pink pigmentation. It was first described in 1993 [1]. Most *Roseomonas species* are environmental bacteria, but some species have been isolated from the skin [2]. The major pathogens in humans are *Roseomonas gilardii*, *Roseomonas cervicalis*, and *Roseomonas mucosa* (*R. mucosa*) [2–4].

Reports of *R. mucosa* infection in humans are limited; however, there have been reports of catheter-related bloodstream infections [5–14] and infections in immunocompromised patients. Cases of bacteremia [4–18], peritonitis [18–21], meningitis [22], infection of soft tissues [4], spinal epidural abscess [23], pyogenic spondylitis [16], cholangitis [11], subretinal abscess [24], infection of the root canal [25], and liver abscess [26] have also been reported. *R. mucosa* is resistant to broad-spectrum cephalosporins [2, 3].

Many patients in these reports were immunocompromised [5–15, 17–21, 24] or had catheter-related [5–14] or postoperative infections [16, 22, 23], with few reports of infection in patients without these characteristics [26]. Reports of skin and soft tissue infections are also rare. Herein, we report a case of cellulitis caused by *R. mucosa* in a non-immunocompromised pediatric patient.

## Case presentation

A 10-year-old boy with no history of immunosuppression had comorbidities, including allergic rhinitis and sebum-deficient eczema, which was treated with topical dexamethasone propionate ointment 0.1%. A few days before the visit, he had been bitten by an insect on the anterior surface of his right lower leg and had scratched it due to itching. The day before the visit, redness and mild pain were observed in the same area, and the patient visited the pediatric department of this hospital.

The vital signs at the time of the patient’s visit to our hospital were as follows: body temperature, 36.1 °C; pulse, 79 beats/minute; and saturation of percutaneous arterial oxygen, 97% (room air). The patient was 140.0 cm tall, weighed 38.0 kg, and had erythema, heat sensation, and swelling with indistinct margins on the anterior aspect of the right lower leg measuring 7 × 7 cm in diameter, with mild pain and itching in the same area. In the center of the erythema, a crust of about 3 × 1 mm and a small amount of pus was observed. Gram staining of the pus showed Gram-negative short bacilli. The right inguinal lymph node was not palpable.

**Fig. 1:**
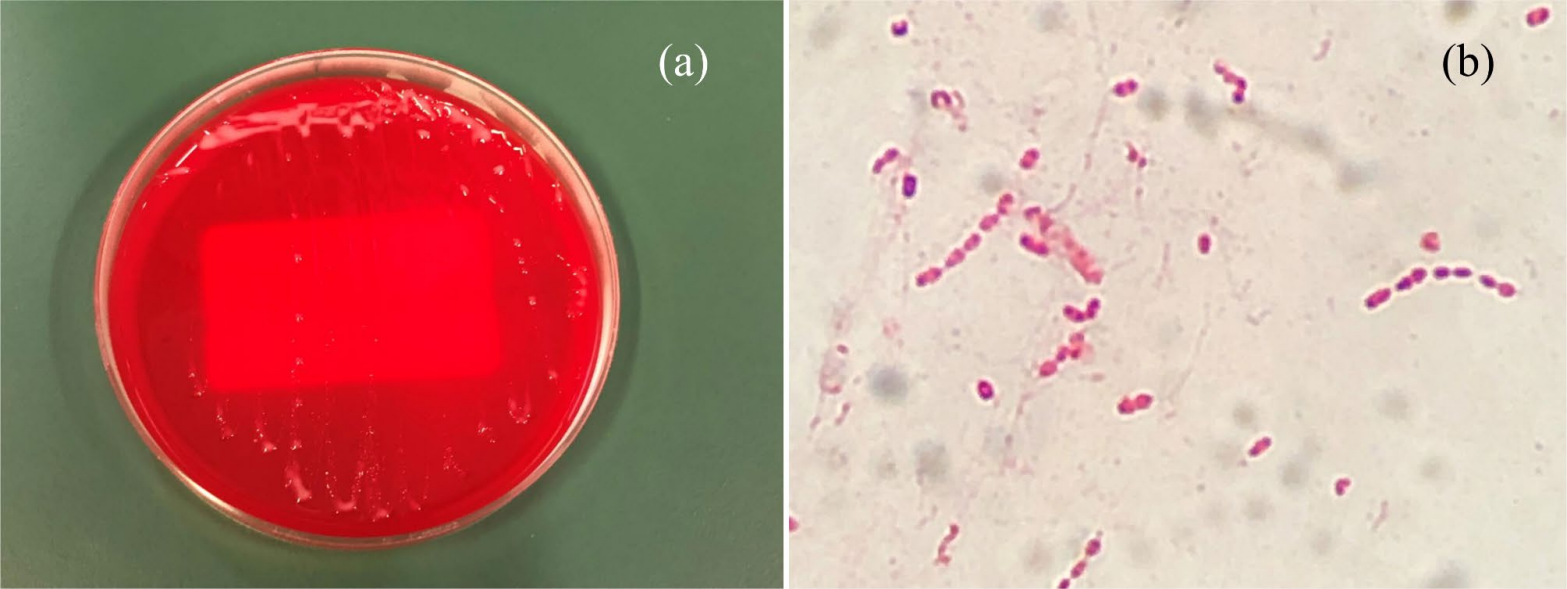
Pink colonies on the blood agar plate (**a**) and Gram stain (**b**)

Based on the symptoms and findings, he was diagnosed with right lower leg cellulitis. The patient had a Gram-negative short bacillus, which was atypical for cellulitis, unlike *Staphylococcus aureus* and *Streptococcus pyogenes*, usually associated with cellulitis (Fig. 1). The patient was treated with oral sulfamethoxazole/trimethoprim and topical betamethasone valerate/gentamicin hydrochloride ointment 0.12%. Because the pruritus was severe, topical betamethasone was used in addition to topical antibiotics. After 3 days, the area of erythema was reduced to approximately 3 × 1 cm, and the pain and itching disappeared. Since the symptoms had largely improved, sulfamethoxazole/trimethoprim was taken orally for 5 days, and the symptoms resolved completely. Cultures of pus detected two types of bacteria, but normal culture methods could not identify one type of bacteria. The sample was analyzed using a Matrix Assisted Laser Desorption/Ionization Time of Flight Mass Spectrometer (MALDI-TOS MS) and identified as *R. mucosa* (score = 2.52). The other bacterium was *Coagulase-Negative Staphylococcus*. Gram staining showed that it was a Gram-negative bacillus. We considered *R. mucosa* to be the causative bacterium.

However, the symptoms disappeared when the bacterium was identified as *R. mucosa*. Susceptibility tests were performed by the broth microdilution method based on standards reported by the Clinical and Laboratory Standards Institute (Table 1). Susceptibility testing for sulfamethoxazole/trimethoprim was not performed. No additional treatment was given because the patient was clinically symptom-free. The patient was followed up until one month after onset, and no relapse of symptoms was observed.

**Table 1:** Results of drug sensitivity test for *Roseomonas mucosa*

Antibiotic	MIC (µg /ml)	Sensitivity
Piperacillin	>64	R
Ceftazidime	>16	R
Cefepime	>16	R
Cefozopran	>16	R
Imipenem/cilastatin	≤1	S
Meropenem	≤1	S
Aztreonam	>16	R
Sulbactam/cefoperazone	>32	R
Tazobactam/piperacillin	>64	R
Gentamicin	≤2	S
Tobramycin	≤ 2	S
Amikacin	≤8	S
Minocycline	≤2	S
Fosfomycin	>16	R
Ciprofloxacin	≤0.5	S
Levofloxacin	≤1	S

The “S” and “R” represent “susceptible” and “resistant,” respectively

## Discussion and conclusions

We encountered a case of cellulitis caused by *R. mucosa*. Since *R. mucosa* exists in soil and moist environments and is a type of resident skin fungus, it rarely causes infections.

Reports of infections caused by *R. mucosa* are limited, and a comprehensive literature review was conducted. A PubMed search using the keyword “*Roseomonas mucosa*” yielded 44 articles, 5 related to skin or soft tissue infections (PubMed was last searched on May 20, 2023). One study reported soft tissue infection in an 8-month-old boy with spinal cord engagement syndrome. He was treated with vancomycin and cefotaxime and was cured [4]. Another case involved the detection of *R. mucosa* in tattoo ink sold in the U.S. [27]. Topical administration of *R. mucosa* from healthy volunteers to patients with atopic dermatitis was reported to improve symptoms [28–30].

Although whole-genome sequencing is the appropriate method for detecting *R. mucosa*, MALDI-TOF MS or 16 S rRNA gene analysis can identify the bacteria for easy and reliable detection in the laboratory. VITEK 2 (bioMérieux, Germany) incorrectly identified this bacterium as *Roseomonas gilardii* or *Rhizobium radiobacter*. However, MALDI-TOF MS and 16 S rRNA genetic analysis reliably identified the species [31]. *R. mucosa* is consistently susceptible to amikacin and imipenem, frequently susceptible to ciprofloxacin and ticarcillin, and essentially resistant to ampicillin, ceftazidime, and cefepime. There are also reports of imipenem/cilastatin-resistant strains [10]; however, its susceptibility to ceftriaxone and sulfamethoxazole/trimethoprim has limited reports [3]. In this case, the patient was resistant to penicillin and cephalosporins but susceptible to carbapenems, quinolones, and aminoglycosides (Table 1). Despite its clinical validity, the susceptibility to sulfamethoxazole/trimethoprim was not tested due to contractual constraints with an outside laboratory.

*R. mucosa* has been frequently reported as an opportunistic infection in immunocompromised patients [5–15, 17–21, 24] and those with catheter-related bloodstream [5–14] or postoperative [16, 22, 23] infections. In the present case, the skin barrier function was originally impaired due to sebum-deficient eczema, and the local immunosuppressive effects caused by topical steroids were assumed to have contributed to an increased susceptibility to infection. In addition, he reported a history of playing in an open and wet area beside his house and was possibly infected by the insects in that area.

In microbiome studies, the area of the skin harboring Gram-negative bacteria overlaps with the area most affected by atopic dermatitis. Bacterial carriage in this region is significantly reduced in patients with atopic dermatitis. Topical administration of *R. mucosa* has been reported to improve symptoms. However, the *R. mucosa* used for topical administration is a specific strain isolated from healthy volunteers, and animal studies using *R. mucosa* derived from atopic patients have reported no change or worsening of local symptoms [28]. We believe that the reason *R. mucosa* did not cause cellulitis or erysipelas in previous reports is that *R. mucosa* was administered from healthy volunteers. However, if the *R. mucosa* was obtained from a patient with a compromised skin barrier function, as in the present case, cellulitis may have occurred.

When skin infections such as cellulitis are observed in patients with compromised skin barrier function, as in this case, or in patients who regularly use topical steroids, one should be aware of rare organisms that can cause opportunistic infections. In particular, when bacteria other than Gram-positive cocci are detected using Gram staining, attention should be paid to drug-resistant bacteria such as *R. mucosa*.

This study has some limitations. First, as this is a case report, it is difficult to generalize the findings to other pediatric patients with hypodermic eczema. Furthermore, the long-term health effects of *R. mucosa* infection remain unknown.

In conclusion, we report a rare case of cellulitis caused by *R. mucosa*. In patients with compromised skin barrier function, regular topical steroid use, and non-Gram-positive cocci detected by Gram staining, it is also necessary to pay attention to infectious diseases caused by rare opportunistic organisms, including *R. mucosa*.

## Data Availability

All data generated or analyzed during this study are included in this published article.

## Abbreviations

*R. mucosa*: *Roseomonas mucosa*
MALDI-TOS MS: Matrix-Assisted Laser Desorption/Ionization Time of Flight Mass Spectrometer
